# Elevating orthopedic documentation: a clinical audit of orthopaedic operative note quality against RCSE and BOA standards

**DOI:** 10.3389/fsurg.2026.1704348

**Published:** 2026-06-10

**Authors:** Ajay Kamat, Ashvath Arumugam Pillai, Upasna Ajmani, Sai Pasya

**Affiliations:** 1 International Training Fellow T&O, NHS Grampian, Aberdeen, United Kingdom; 2 SSPM Medical College and Lifetime Hospital, Kudal, Sindhudurg, India; 3 LAT 3 Ophthalmology, NHS Grampian, Aberdeen, United Kingdom; 4 University of Kansas Medical Center, Kansas City, KS, United States

**Keywords:** clinical audit, operative notes, orthopedic audit, quality improvement, RCSE/BOA standards

## Abstract

**Background:**

The main aim of this audit was to measure the performance of the orthopedic Operating Theatre (OT) notes as compared to the standards laid down by the Royal College of Surgeons of England (RCSE), British Orthopedic Association (BOA) and their collaborators.

**Methods:**

This single-cycle audit was conducted in the Department of Orthopaedics of a tertiary care hospital. One hundred randomly selected orthopaedic operative theatre notes from January–June 2023 were assessed against RCSE and BOA documentation standards. Following identification of deficiencies, targeted interventions were implemented between July–October 2023, comprising a standardised documentation template and a staff training programme. A re-audit of 100 notes was subsequently performed from October 2023–March 2024 to evaluate the impact of these interventions. Domain-specific compliance rates were compared between cycles using the chi-square test.

**Results:**

Baseline audit identified deficiencies across four documentation domains: patient identification (72%), intraoperative findings (65%), postoperative plan (58%), and surgeon details (70%). Following the intervention programme, re-audit demonstrated significant improvements in all domains: patient identification (95%, *p* < 0.001), intraoperative findings (95%, *p* < 0.001), postoperative plan (90%, *p* < 0.001), and surgeon details (97%, *p* < 0.001). Overall protocol adherence improved from 68% to 95% (*p* < 0.001). Persistent deficiency was noted in completeness of postoperative plan documentation (10% non-compliance at re-audit).

**Conclusion:**

A single audit cycle with targeted training and a standardised template produced statistically significant improvements across all RCSE/BOA documentation domains. Incorporating operative note training into routine induction programmes for rotating staff is recommended to sustain these gains.

## Introduction

Operative notes are critical medical records, and maintaining them accurately is the responsibility of every surgeon (1). It is crucial for patient safety, continuity of care, and medico-legal obligations to have accurate and comprehensive OT notes (2). The RCSE and BOA have provided guidelines on what OT documentation needs to be included in OT medical records (2, 3). Despite these guidelines, deficits in OT notes are frequent and can compromise both patient outcomes and medico-legal defense (4, 5).

The surgical teams and the critical care teams post-operatively analyze the operative notes as the first point of contact with any formal documentation to understand the patient under their review (6, 7). Healthcare professionals are guided regarding the details of the procedure as well as essential post-operative care that the patient requires (8, 9). Thromboprophylaxis and antibiotics are decided by the surgeon in-charge post-operatively. These critical decisions are made by surgeons on a case-to-case basis (10, 11). The post-operative team often seeks guidance regarding such management in the post-operative notes. Hence, the quality of operative notes is directly related to the effective management of the surgical patient (6, 12).

## Aim

1. To determine the quality of orthopaedic operating theatre notes against RCSE and BOA guidelines during the baseline assessment, identifying deficiencies across the 18 essential documentation parameters (2, 3)
2. To evaluate the effects of targeted interventions on the quality of OT documentation during the re-audit (13, 14)

## Materials and methods

The audit was conducted as a single audit cycle comprising a baseline assessment and a re-audit:

- **Baseline Assessment (January 2023–June 2023)**: Baseline data was collected, looking at the quality of OT notes and the extent of meeting RCSE and BOA guidelines (13).
- **Re-Audit (October 2023–March 2024)**: Follow-up audit after a series of interventions, including staff training and standardized templates, to measure improvements in the quality of documentation (13–15).

### Inclusion and exclusion criteria

Inclusion criteria: all orthopaedic operating theatre notes documented during the respective audit periods. Exclusion criteria: (1) operative notes from departments other than orthopaedics; (2) notes with insufficiently completed data that precluded meaningful assessment.

Each operative note was assessed against 18 essential parameters specified in the RCSE and BOA guidelines, grouped into four domains: (i) **patient identification**—patient name, date of birth, and hospital number; (ii) **intraoperative findings**—operative diagnosis, procedure performed, incision used, intraoperative findings, complications, implant details, and closure technique; (iii) **postoperative plan**—postoperative instructions, thromboprophylaxis plan, and antibiotic plan; (iv) **surgeon details**—operating surgeon name, assistant name, grade, GMC number, and signature. Each parameter was recorded as either present or absent. Overall compliance was defined as the presence of all required parameters within a given domain.

This audit was conducted in the Department of Orthopaedics of a tertiary care hospital. The department performs orthopaedic surgical procedures across trauma and elective subspecialties. The surgical team comprises consultant orthopaedic surgeons, specialty trainees, and rotating Resident Medical Officers (RMOs).

### Data collection

One hundred OT notes were selected randomly from orthopaedic cases conducted from January—June 2023. Each note was assessed independently against a standardised checklist of 18 essential parameters derived from RCSE and BOA guidelines, grouped into four domains: (i) patient identification, (ii) intraoperative findings, (iii) postoperative plan, and (iv) surgeon details (4, 7, 9). Discrepancies between assessors were resolved by consensus. An identical methodology was applied to 100 randomly sampled notes for the re-audit (October 2023–March 2024) (15).

### Statistical analysis

Compliance rates for each documentation domain were expressed as percentages. Chi-square tests (without Yates' continuity correction) were used to compare compliance rates between the baseline and re-audit cycles for each domain. A *p*-value of <0.05 was considered statistically significant. All analyses were performed using R (version 4.3.2).

#### Training

The study documented a range of deficiencies and omissions & highlighted the need for a focused intervention program aimed at improving the quality of OT notes (4, 16). The intervention consisted of a training program and a new standardized documentation form developed in accordance with guidelines provided by the Royal College of Surgeons of England (RCSE) and the British Orthopedic Association (BOA) (2, 3). The purpose of the training program was to provide education to the surgical team (orthopedic surgeons, residents, and OT staff) regarding the potential significance of accurate and thorough documentation of OT notes (11, 17). It was conducted from July 2023–October 2023.

The first training program module addressed the legal, ethical, and clinical importance of accurate OT note documentation based on real-life case studies (18, 19). The next training sessions covered RCSE and BOA standards to address areas requiring improvement in the audit (2, 3).

After the training slides were completed, staff was asked to participate in the mock documentation of a standardized surgical case using the new documentation tool (20, 21). Feedback was solicited in real time to help demonstrate some of the challenges encountered (22).

Participants were invited to voice any challenges faced, or issues with understanding, during training sessions. This led to a more dynamic training approach in which trainers were able to address issues and adapt the training objectives to fit the surgical team needs (23, 24).

Subsequent to the training, evaluations were conducted to assess the efficacy of the training program as well as any areas of content or practical application that were still lacking. Evaluations consisted of quizzes, practical tasks, as well as peer evaluations (11, 22).

### Protocol implementation

**Figure 1:**
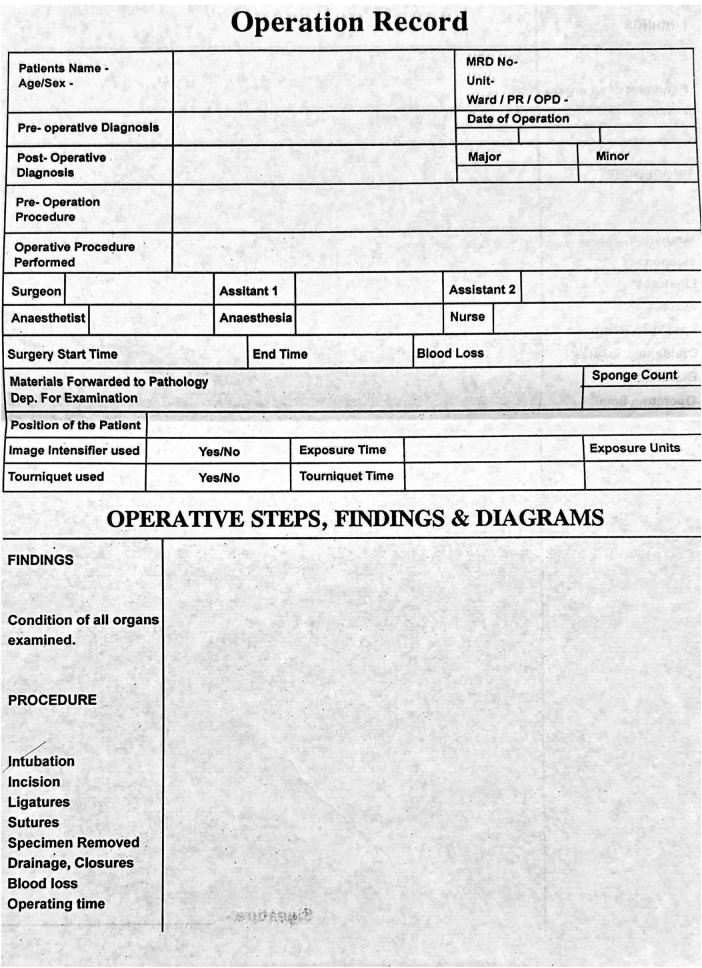

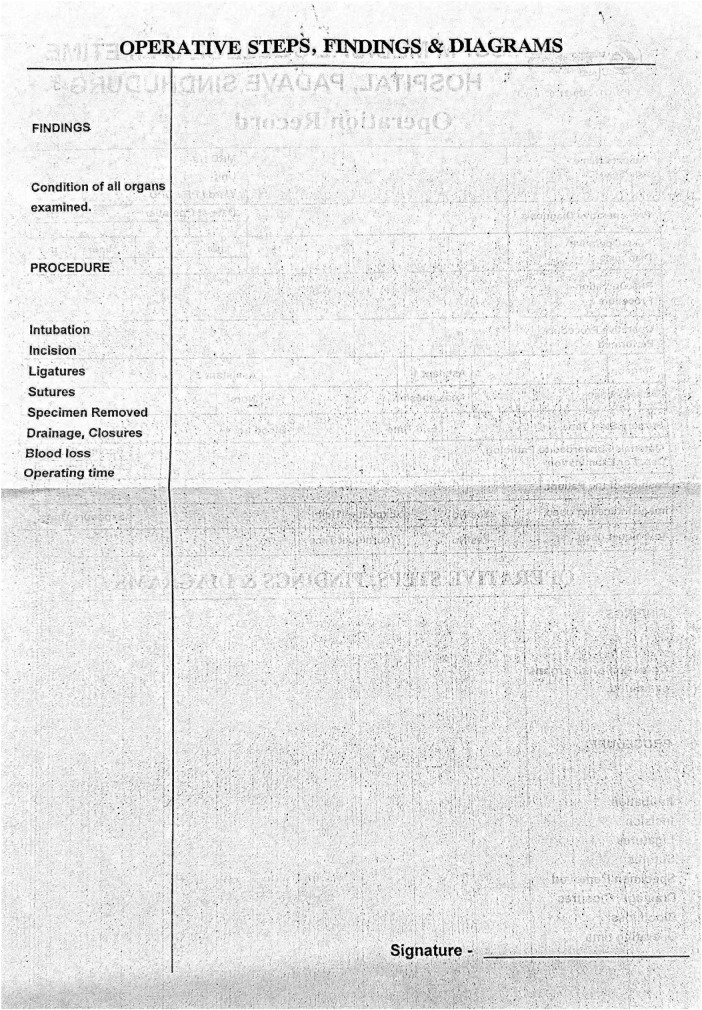
Old template.

Prior to the intervention, operative notes were documented using an unstructured format (Figure 1).

**Figure 2:**
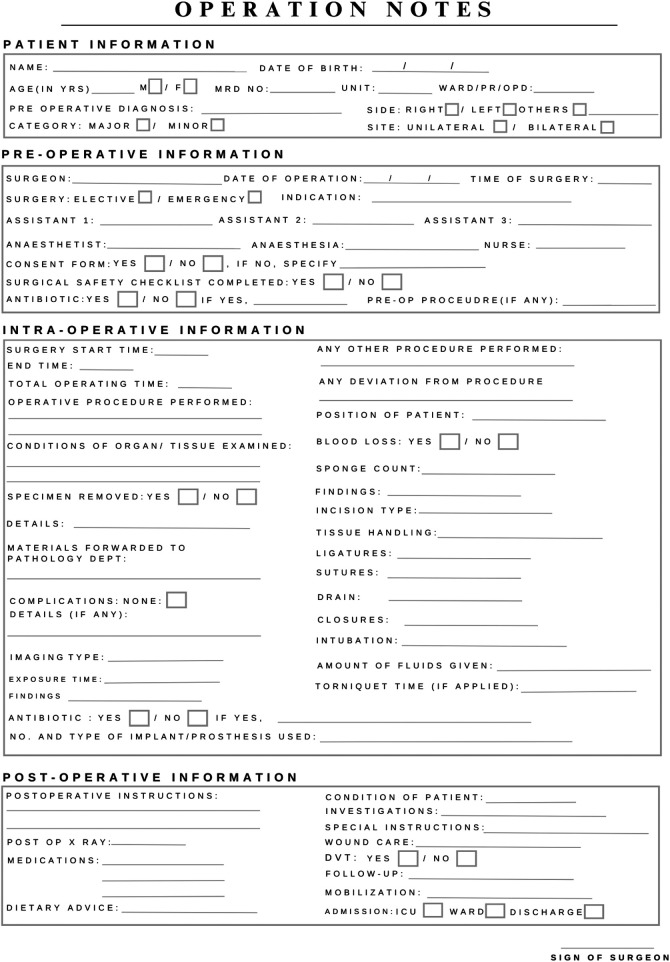
New standardised documentation form.

A standardized template for OT notes was developed, ensuring consistent documentation for each orthopedic procedure (Figure 2) (8). The template intended to include all elements of documentation required by RCSE and BOA standards in an easy-to-follow format that captured patients' particulars, procedure details, intraoperative findings and the postoperative plan (2, 3).

The template was standardized; however, it allowed for variability about different types of orthopedic surgeries (8, 11). In addition, prompts were included, directing the surgeon to be succinct and convey as much detail as possible relevant to the type of surgery (8).

The template was piloted on a small segment of surgeries, then comments from the pilot were assessed to ensure the template documentation was both easy and of sufficient detail (8–21).

The standardized template was made mandatory across OT notes in Orthopedics (8). Adherence was monitored for conformance and any cases of non-compliance were corrected (22). The supporting materials were provided to help the surgeons in the transition including laminated guideline cards of the template elements and availability of linked examples of well-written OT notes (21).

To ensure that surgeons and the OT staff would have continued support, documentation mentors were assigned and available during the initial transition period (11, 22).

As the template was instituted, regular audits were placed to determine continued compliance with the template and investigate other issues (13, 14). Continuous systems of feedback were established by sharing the audit findings with the surgical and OT staff, leading to both continuous improvement for the training program and template documentation (22, 24).

## Results

### Baseline audit findings (January–June 2023)

**Figure 3:**
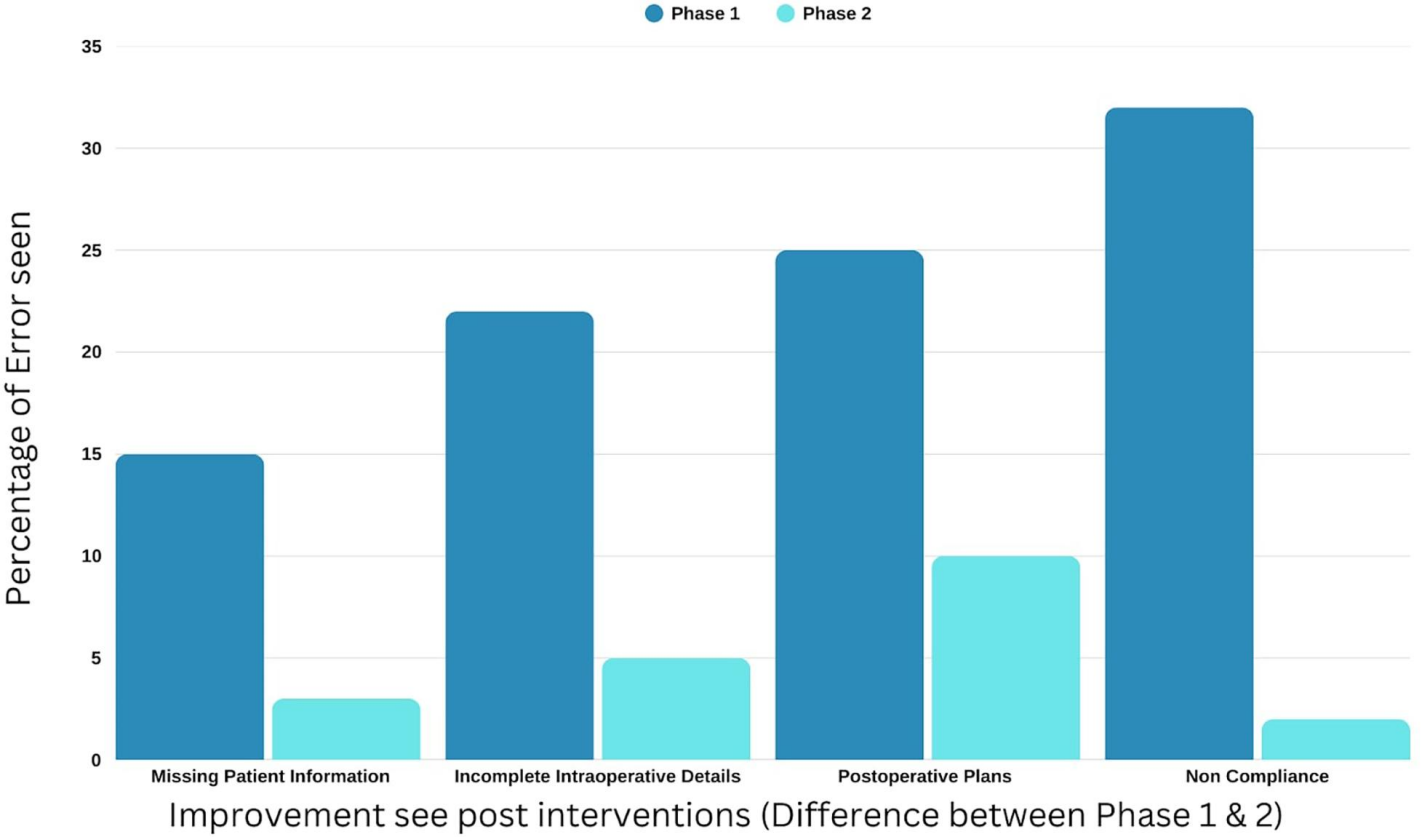
Comparison of documentation compliance rates across domains between baseline audit (January–June 2023) and re-audit (October 2023–March 2024) [“Phase 1” → “baseline (2023)” and “Phase 2” → “Re-audit (2024)”].

One hundred orthopaedic operative notes were assessed against 18 RCSE/BOA parameters across four domains (Figure 3). Deficiencies were identified across all domains: patient identification 72%, intraoperative findings 65%, surgeon details 70%, and postoperative plan 58%—the most deficient domain. Overall protocol adherence was 68% (11, 21). Table 1 summarises compliance rates across all domains for both audit cycles.

**Table 1:** Compliance rates across documentation domains at baseline and re-audit, with chi-square statistics.

Documentation domain	Baseline (*n* = 100)	Re-audit (*n* = 100)	*χ* ^2^	*p*-value
Patient Identification	72%	95%	19.20	<0.001
Intraoperative Findings	65%	95%	28.12	<0.001
Postoperative Plan	58%	90%	26.61	<0.001
Surgeon Details	70%	97%	26.46	<0.001
Overall Adherence	68%	95%	24.18	<0.001

### Re-audit findings (October 2023–March 2024)

Following the intervention programme, re-audit demonstrated statistically significant improvements across all domains (Table 1, Figure 3): patient identification improved from 72% to 95% (*χ*^2^ = 19.20, *p* < 0.001), intraoperative findings from 65% to 95% (*χ*^2^ = 28.12, *p* < 0.001), postoperative plan from 58% to 90% (*χ*^2^ = 26.61, *p* < 0.001), and surgeon details from 70% to 97% (*χ*^2^ = 26.46, *p* < 0.001). Overall protocol adherence improved from 68% to 95% (*χ*^2^ = 24.18, *p* < 0.001). Postoperative plan documentation remained the area of greatest persistent deficiency, with 10% of notes still lacking complete clarity (4, 7). Following the intervention programme, re-audit demonstrated statistically significant improvements across all domains (Figure 3).

After implementing the OT note template, adherence to the new documentation protocol increased from 68% in 2023 to 95% in 2024, which was a 27% improvement. This indicates that staff was not only engaged but also has adopted the standardized OT note template (20).

The feedback mechanism was shown to be highly effective as 92% of surgeons reported the feedback they received following each audit cycle was useful for improving their documentation practices, with modifications in subsequent audit periods leading to a consistent compliance rate of 90% or higher in 94% of the critical documentation category (22, 24).

The improved documentation practices facilitated continuity of care, with 88% of the healthcare team indicating the facilitated transition of care, in response to improved detail and reliable consistency of OT notes (6, 7). During the re-audit period, incident reports attributable to communication failures related to operative documentation—including missed thromboprophylaxis orders, incorrect antibiotic prescriptions, and ambiguous wound care instructions—decreased by 25% compared with the baseline period, as recorded in the hospital incident reporting system. It is acknowledged that this association is observational; causation cannot be established without a controlled study design, and concurrent changes in clinical practice cannot be excluded as contributory factors (12).

Post-intervention survey implemented among the surgical team in 2024 resulted in 85% of respondents indicating they felt the interventions had positively influenced their approach to documentation. That cultural shift is evident with sustained high compliance rates and the quality of OT notes (22).

In addition, with 90% of newly inducted surgeons and OT staff in 2024 stating they were familiar and comfortable with the standardized template in their first month of practice, the impact of the intervention on the practice is expected to be a lasting benefit for the department (11, 22).

## Discussion

With the increasing number of medicolegal cases in recent years, documentation around the perioperative period is required to be accurate and legible to provide a clear record of events (18). The General Medical Council (GMC) recommends that operative documentation is as accurate, comprehensive, and legible as possible as part of the Good Medical Practice Guidance (19).

In our study, patient identification improved to 95%, which is in accordance with a study conducted by Hasan R et al. in 2023 where improvement was noted in 97% of the operative notes in the re-audit cycle (24). Detailed intraoperative findings were mentioned in 95% of OT notes in the re-audit conducted by us, which is in line with a study done by Nasim O et al. in 2022 where the percentage raised to 95.89% at the registrar level (6).

Detailed postoperative documentation was still lacking in 10% of the notes; this could be due to the fact that there are faculty in this hospital who are on a visiting basis and it was difficult to always secure their presence in the training sessions (23). From a logistic standpoint, the changing shifts of Resident Medical Officers and the regular recruitment of new healthcare personnel compounds this challenge further (17, 23).

One practical strategy to address this logistical challenge is to incorporate operative note documentation training as a mandatory component of the routine induction programme for all newly appointed doctors and rotating staff (23). Standardised induction-based training would ensure that every incoming clinician—regardless of rotation frequency—is oriented to RCSE/BOA standards and the departmental template prior to their first operative case, reducing dependence on *ad hoc* training sessions that visiting or rotating staff frequently miss (17, 23).

In general, 95% of OT notes adhered to the standardized template, demonstrating a high level of compliance with the new protocols (20). This standardization has significantly improved the overall quality of OT documentation, making it easier to audit and ensuring that all critical information is consistently captured (4, 11).

While two distinct interventions were implemented—a structured training programme and a standardised documentation template—their combined effect was evaluated through a single composite measure of domain-specific compliance, as separating their individual contributions was not feasible within the audit design. Future studies could employ a controlled design to isolate the effect of each intervention independently.

### Continuing challenges and recommendations

Generalizability is a concern! As this study evaluates the impact of a new proforma of operative notes in a single tertiary care hospital, it may not be applied to each and every hospital (16, 23). It also might not consider concurrent changes in clinical practices and patient outcomes. Emphasis on quantitative parameters might overlook qualitative aspects of documentation quality.

This audit sheds light on opportunities to enhance surgeons' documentation practices in the future. In the course of this audit cycle, it was observed that assessing practices against established standards and subsequent re-auditing resulted in a substantial improvement, aligning with findings reported by other researchers (25–27).

Legibility of handwritten notes still remains a question! Research consistently supports the effectiveness of computer templates or typed notes over their handwritten counterparts (28). Due to financial constraints, our hospital has yet to adopt electronic operation notes. Nevertheless, there is a firm conviction that electronic note documentation represents the pinnacle of legibility standards (10, 29).

Despite the improvement, 10% of OT notes still lacked complete clarity of postoperative plans. Further training sessions should focus on this area, perhaps with case-based discussions that highlight the consequences of vague postoperative instructions (6, 9).

Although protocol adherence was high, continuous monitoring and periodic retraining are essential to maintain these standards (22). A quarterly review of OT notes should be implemented, with feedback provided to surgeons to ensure sustained improvement (24).

The standardized template is effective for routine procedures, but some complex cases may require additional documentation elements (20). Developing supplementary guidelines for such cases could further enhance documentation quality (21).

## Conclusion

This single-cycle audit demonstrated that a structured intervention—comprising a standardised RCSE/BOA-aligned documentation template and a targeted staff training programme—produced statistically significant improvements across all four operative note domains in the Orthopaedic Department (2–4, 11, 20). Overall protocol adherence improved from 68% at baseline to 95% at re-audit (*p* < 0.001). Postoperative plan documentation remains the most persistently deficient domain, with 10% non-compliance at re-audit, and represents the priority area for continued quality improvement efforts (22, 24). Incorporating operative note documentation training into routine induction programmes for rotating and newly appointed staff is recommended as a practical and sustainable intervention. The structured methodology described—comprising baseline assessment, targeted intervention, and re-audit—is reproducible and may serve as a model for clinical audit in other surgical specialties (15, 24).

## Data Availability

The dataset generated during this audit is fully anonymized and does not contain any patient-identifiable information. However, due to institutional policy, raw audit datasets are not publicly deposited. Aggregated data supporting the findings are included in the manuscript, and further details may be obtained from the corresponding author upon reasonable request. Requests to access the datasets should be directed to Ajay Kamat, ajayvkamat@gmail.com.
